# Crystal structures of ZnCl_2_·2.5H_2_O, ZnCl_2_·3H_2_O and ZnCl_2_·4.5H_2_O

**DOI:** 10.1107/S1600536814024738

**Published:** 2014-11-15

**Authors:** Erik Hennings, Horst Schmidt, Wolfgang Voigt

**Affiliations:** a TU Bergakademie Freiberg, Institute of Inorganic Chemistry, Leipziger Strasse 29, D-09596 Freiberg, Germany

**Keywords:** crystal structure, low-temperature salt hydrates, chloride hydrates, zinc salts

## Abstract

The formation of different complexes in aqueous solutions is an important step in understanding the behavior of zinc chloride in water. The structure of concentrated ZnCl_2_ solutions is governed by coordination competition of Cl^−^ and H_2_O around Zn^2+^. According to the solid–liquid phase diagram, the title compounds were crystallized below room temperature. The structure of ZnCl_2_·2.5H_2_O contains Zn^2+^ both in a tetra­hedral coordination with Cl^−^ and in an octa­hedral environment defined by five water mol­ecules and one Cl^−^ shared with the [ZnCl_4_]^2−^ unit. Thus, these two different types of Zn^2+^ cations form isolated units with composition [Zn_2_Cl_4_(H_2_O)_5_] (penta­aqua-μ-chlorido-tri­chlorido­di­zinc). The trihydrate {hexa­aqua­zinc tetra­chlorido­zinc, [Zn(H_2_O)_6_][ZnCl_4_]}, consists of three different Zn^2+^ cations, one of which is tetra­hedrally coordinated by four Cl^−^ anions. The two other Zn^2+^ cations are each located on an inversion centre and are octa­hedrally surrounded by water mol­ecules. The [ZnCl_4_] tetra­hedra and [Zn(H_2_O)_6_] octa­hedra are arranged in alternating rows parallel to [001]. The structure of the 4.5-hydrate {hexa­aqua­zinc tetra­chlorido­zinc trihydrate, [Zn(H_2_O)_6_][ZnCl_4_]·3H_2_O}, consists of isolated octa­hedral [Zn(H_2_O)_6_] and tetra­hedral [ZnCl_4_] units, as well as additional lattice water mol­ecules. O—H⋯O hydrogen bonds between the water mol­ecules as donor and ZnCl_4_ tetra­hedra and water mol­ecules as acceptor groups leads to the formation of a three-dimensional network in each of the three structures.

## Abstract 2

The crystal structures of ZnCl_2_·*x*H_2_O (x = 2.5, 3 and 4.5) consist of Zn^2+^ ions both in an octa­hedral and tetra­hedral environment. O—H⋯O hydrogen bonds between water mol­ecules and tetra­hedral ZnCl_4_ units lead to the formation of a three-dimensional network in each of the structures.

## Chemical context   

Zinc chloride solutions, especially at lower temperatures, are helpful in the understanding of the formation of different complex ion species in solution. The solubility of zinc chloride in water has been investigated by several authors in different concentration areas and at different temperatures (Haghighi *et al.*, 2008 ▶; Mylius & Dietz, 1905 ▶; Jones & Getman, 1904 ▶; Chambers & Frazer, 1900 ▶; Biltz, 1902 ▶; Dietz, 1899 ▶; Etard, 1894 ▶). In the literature (Mylius & Dietz, 1905 ▶), the 4-, 3-, and 2.5-hydrates have been reported at lower temperatures. We have also found the 2.5-hydrate, the trihydrate and the 4.5-hydrate as stable phases along the equilibrium crystallization curves. The 4.5-hydrate crystallizes below 240 K. The crystal structure of the trihydrate reported herein has also been determined by Wilcox (2009 ▶) in his thesis, but was never published. While writing the formula of the trihydrate in a more detailed formula as [Zn(H_2_O)_6_][ZnCl_4_], the analogy to other structures like that of [Mg(H_2_O)_6_][SO_4_] (Zalkin *et al.*, 1964 ▶) and [Zn(H_2_O)_6_][SO_4_] (Spiess & Gruehn, 1979 ▶) becomes obvious. These structures are very similar in the arrangement of octa­hedral units and anions in the unit cell.

## Structural commentary   

**Figure 1:**
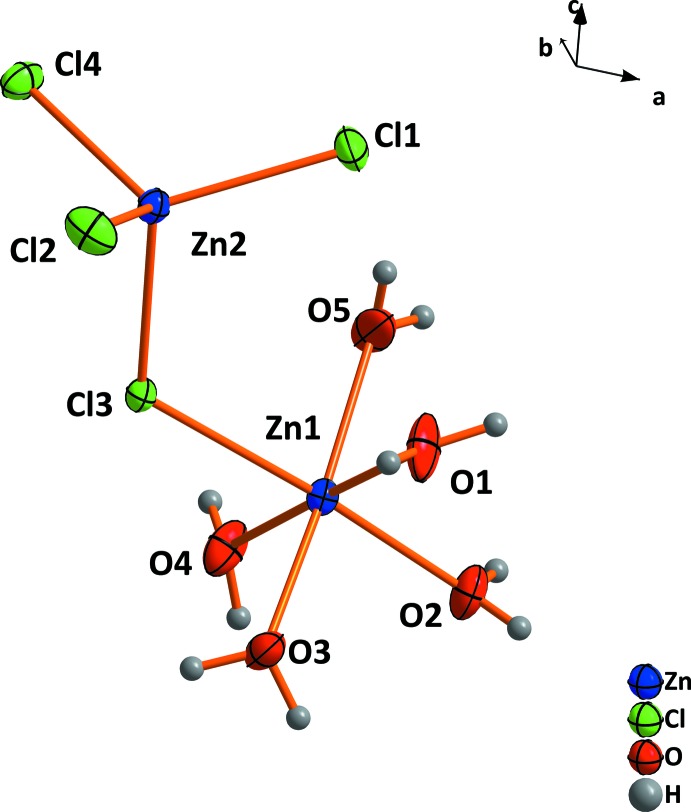
The asymmetric unit of ZnCl_2_·2.5H_2_O. Displacement ellipsoids are drawn at the 50% probability level.

**Figure 2:**
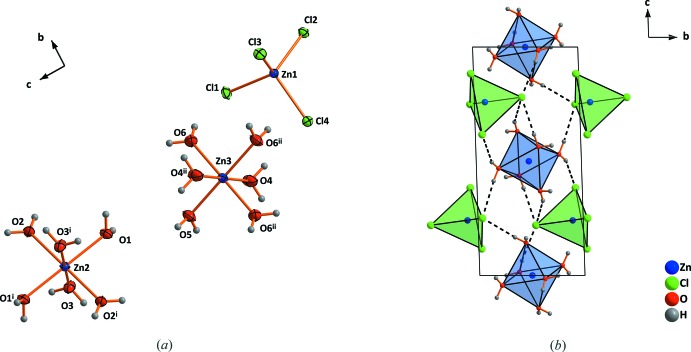
(*a*) The mol­ecular units and (*b*) the unit cell in the structure of ZnCl_2_·3H_2_O. Displacement ellipsoids are drawn at the 50% probability level. Dashed lines indicate hydrogen bonds. [Symmetry codes: (i) 1 − *x*, 1 − *y*, 2 − *z*; (ii) 1 − *x*, 1 − *y*, 1 − *z*.]

**Figure 3:**
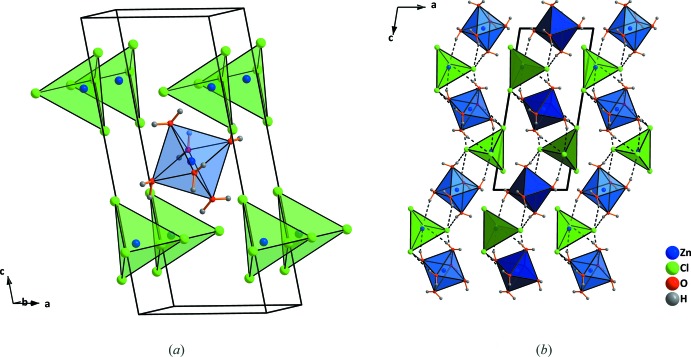
(*a*) Arrangement of [ZnCl_4_]^2−^-anions and [Zn(H_2_O)_6_]^2+^ cations in a CsCl-like structure and (*b*) formation of chains by alternation of different coordination polyhedra in ZnCl_2_·3H_2_O. Dashed lines indicate hydrogen bonds. Only hydrogen bonds in one chain are shown.

**Figure 4:**
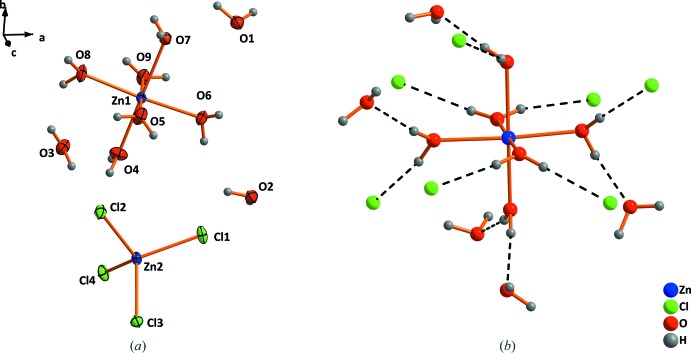
(*a*) The mol­ecular units in the structure of ZnCl_2_·4.5H_2_O and (*b*) formation of a second coordination shell. Displacement ellipsoids are drawn at the 50% probability level. Dashed lines indicate hydrogen bonds.

Within the crystal structure of the 2.5-hydrate, there are two crystallographic different Zn^2+^ cations, as shown in Fig. 1 ▶. The Zn1 cation is octa­hedrally coordinated by five water mol­ecules and one chloride anion. The Zn2 cation is coordinated by four chloride anions, one shared with the Zn1 cation, leading to the formation of isolated [Zn_2_Cl_4_(H_2_O)_5_] units. Since the bond lengths of the bridging Cl atom of the tetra­hedron are shorter than to that of the octa­hedron, the latter becomes more distorted. The crystal structure of zinc chloride trihydrate consists of three crystallographically different Zn^2+^ cations (Fig. 2 ▶ *a*). Two (Zn2 and Zn3) are located about an inversion centre and are coordinated octa­hedrally by six water mol­ecules, forming [Zn(H_2_O)_6_]^2+^ cations. The third one (Zn1) is tetra­hedrally coordinated by chlorine anions, [ZnCl_4_]^2−^. The polyhedra are not connected by sharing a single atom like in the 2.5-hydrate, but they are linked by hydrogen bonds (Fig. 2 ▶ *b*). The octa­hedra and tetra­hedra are arranged in a CsCl-like arrangement with eight tetra­hedra located around one octa­hedron (Fig. 3 ▶ *a*). As shown in Fig. 4 ▶ *a*, in the asymmetric unit of ZnCl_2_·4.5H_2_O, two different Zn^2+^ cations are present. The Zn1 cation is coordinated octa­hedrally by six water mol­ecules and the Zn2 cation tetra­hedrally by four chloride anions. The three remaining water mol­ecules are hydrogen-bonded to a [Zn1(H_2_O]^2+^ octa­hedron (Fig. 4 ▶ *b*).

## Supra­molecular features   

**Figure 5:**
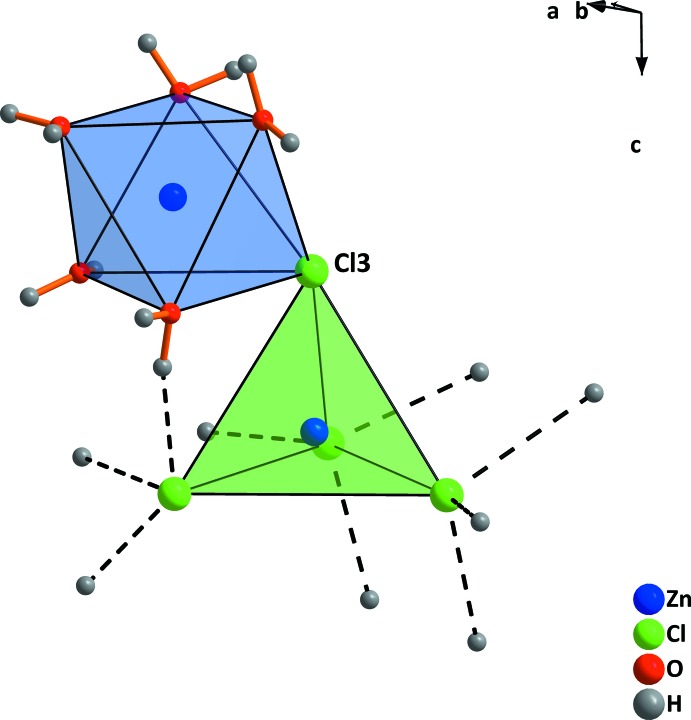
The connection of individual [Zn_2_Cl_4_(H_2_O)_5_] units through hydrogen bonds (dashed lines) in the structure of ZnCl_2_·2.5H_2_O.

In the structure of ZnCl_2_·2.5H_2_O, all terminal Cl^−^ anions are connected to the octa­hedral parts of neighbouring [Zn_2_Cl_4_(H_2_O)_5_] units by three O—H⋯Cl hydrogen bonds per anion (Table 1 ▶, Fig. 5 ▶). The coordination polyhedra in the trihydrate are arranged in zigzag chains parallel to [001] in the crystal structure. The chains are highlighted in different shades of colors in Fig. 3 ▶ *b*. Hydrogen bonds (Table 2 ▶) are established within one chain and between neighbouring chains (not shown in the Figure). As can be seen from Fig. 4 ▶ *b*, five water mol­ecules in the crystal structure of ZnCl_2_·4.5H_2_O are connected *via* hydrogen bonds to the [Zn1(H_2_O]^2+^ octa­hedron, three of them at the axial coordination sites and two of them at the equatorial coordination sites. Seven chloride anions from [Zn2Cl_4_]^2−^ tetra­hedra contribute to the second coordination sphere of Zn1. Thus, every coordinating water mol­ecule forms two hydrogen bonds. The structural situation in this salt can be compared with the second coordination shells around magnesium in magnesium halide nonahydrates like MgBr_2_·9H_2_O or MgI_2_·9H_2_O (Hennings *et al.*, 2013 ▶). Each water mol­ecule of the [Mg(H_2_O)_6_]^2+^ octa­hedra forms two hydrogen bonds, thus six water mol­ecules and six halide atoms are involved in the second shell. However, in case of the magnesium halides each water mol­ecule donates a hydrogen bond towards a halide anion and towards another water mol­ecule. The hydrogen-bond geometry in ZnCl_2_·4.5H_2_O is given inTable 3 ▶.

## Database survey   

For crystal structures of other zinc chloride hydrates (ZnCl_2_·*R*H_2_O), see: Follner & Brehler (1970 ▶; *R* = 1.33); Wilcox (2009 ▶; *R* = 3). For crystal structures of anhydrous zinc chloride, see: Brehler (1961 ▶); Yakel & Brynestad (1978 ▶). For similar structural set-ups in comparison with the 3-hydrate, [Zn(H_2_O)_6_][ZnCl_4_], see: Zalkin *et al.* (1964 ▶; [Mg(H_2_O)_6_][SO_4_]); Spiess & Gruehn (1979 ▶; [Zn(H_2_O)_6_][SO_4_]); Agron & Busing (1985 ▶; [Mg(H_2_O)_6_][Cl_2_]); Ferrari *et al.* (1967 ▶; [Zn(H_2_O)_6_][NO_3_]_2_).

## Synthesis and crystallization   

Zinc chloride 2.5 hydrate was crystallized from an aqueous solution of 73.41 wt% ZnCl_2_ at 280 K after 2 d, zinc chloride trihydrate from an aqueous solution of 69.14 wt% ZnCl_2_ at 263 K after 2 d and zinc chloride 4.5 hydrate from an aqueous solution of 53.98 wt% ZnCl_2_ at 223K after 2 d. For preparing these solutions, zinc chloride (Merck, 99%) was used. The content of Zn^2+^ was analysed by complexometric titration with EDTA. The crystals are stable in their saturated solutions over a period of at least four weeks. The samples were stored in a freezer or a cryostat at low temperatures. The crystals were separated and embedded in perfluorinated ether for X-ray diffraction analysis.

## Refinement   

Crystal data, data collection and structure refinement details are summarized in Table 4 ▶. The H atoms of each structure were placed in the positions indicated by difference Fourier maps. For all three structures, distance restraints were applied for all water mol­ecules, with O—H and H—H distance restraints of 0.84 (1) and 1.4 (1) Å, respectively. For ZnCl_2_·2.5H_2_O *U*_iso_ values were set at 1.2*U*_eq_(O) using a riding-model approximation.

## Supplementary Material

Crystal structure: contains datablock(s) ZnCl2_2halbH2O_150K, zncl2_3H2O_150K, ZnCl2_4halbH2O_120K. DOI: 10.1107/S1600536814024738/wm5081sup1.cif

Structure factors: contains datablock(s) ZnCl2_2halbH2O_150K. DOI: 10.1107/S1600536814024738/wm5081ZnCl2_2halbH2O_150Ksup2.hkl

Structure factors: contains datablock(s) zncl2_3H2O_150K. DOI: 10.1107/S1600536814024738/wm5081zncl2_3H2O_150Ksup3.hkl

Structure factors: contains datablock(s) ZnCl2_4halbH2O_120K. DOI: 10.1107/S1600536814024738/wm5081ZnCl2_4halbH2O_120Ksup4.hkl

CCDC references: 1033587, 1033586, 1033585

Additional supporting information:  crystallographic information; 3D view; checkCIF report

## Figures and Tables

**Table 1:** Hydrogen-bond geometry (, ) for ZnCl_2_2.5H_2_O

*D*H*A*	*D*H	H*A*	*D* *A*	*D*H*A*
O1H1*A*Cl2^i^	0.83(1)	2.43(1)	3.243(2)	167(3)
O1H1*B*O5^ii^	0.84(1)	2.02(1)	2.853(3)	178(4)
O2H2*A*Cl2^iii^	0.83(1)	2.51(2)	3.299(2)	158(3)
O2H2*B*Cl4^ii^	0.84(1)	2.41(1)	3.2212(19)	162(3)
O3H3*B*Cl1^iv^	0.83(1)	2.42(1)	3.225(2)	164(3)
O3H3*A*Cl4^v^	0.83(1)	2.38(1)	3.205(2)	171(3)
O4H4*B*Cl2^v^	0.83(1)	2.35(1)	3.181(2)	177(3)
O4H4*A*Cl1^iii^	0.83(1)	2.45(2)	3.2349(19)	157(3)
O5H5*A*Cl4^i^	0.83(1)	2.55(2)	3.233(2)	141(3)
O5H5*B*Cl1	0.83(1)	2.56(1)	3.359(3)	163(3)

Symmetry codes: (i) 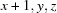; (ii) 
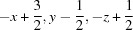
; (iii) 
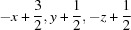
; (iv) 
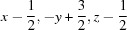
; (v) 
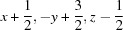
.

**Table 2:** Hydrogen-bond geometry (, ) for ZnCl_2_3H_2_O

*D*H*A*	*D*H	H*A*	*D* *A*	*D*H*A*
O1H1*B*Cl3^i^	0.84(1)	2.42(1)	3.2520(14)	168(4)
O1H1*A*Cl4^ii^	0.84(1)	2.43(1)	3.2431(14)	166(3)
O2H2*A*Cl2^iii^	0.84(1)	2.41(2)	3.2260(14)	163(4)
O2H2*B*Cl3^iv^	0.84(1)	2.54(2)	3.3264(15)	157(3)
O3H3*B*Cl2^ii^	0.84(1)	2.42(2)	3.1715(14)	149(3)
O3H3*B*Cl2^v^	0.84(1)	2.81(3)	3.3159(14)	120(2)
O3H3*A*Cl4^iv^	0.83(1)	2.45(1)	3.2552(15)	162(3)
O4H4*A*Cl4	0.84(1)	2.43(2)	3.2307(18)	159(4)
O4H4*B*Cl1^vi^	0.84(1)	2.39(1)	3.2114(17)	167(4)
O5H5*B*Cl3^vii^	0.84(1)	2.91(5)	3.4565(17)	125(5)
O5H5*B*Cl4^vii^	0.84(1)	2.59(3)	3.3527(18)	151(6)
O5H5*A*Cl1^ii^	0.84(1)	2.48(1)	3.3159(18)	170(5)
O6H6*A*Cl1	0.84(1)	2.52(2)	3.3142(18)	158(4)
O6H6*B*Cl3^i^	0.84(1)	2.41(1)	3.2405(17)	169(3)

Symmetry codes: (i) 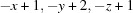; (ii) 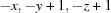; (iii) 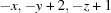; (iv) 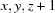; (v) 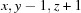; (vi) 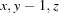; (vii) 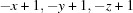.

**Table 3:** Hydrogen-bond geometry (, ) for ZnCl_2_4.5H_2_O

*D*H*A*	*D*H	H*A*	*D* *A*	*D*H*A*
O1H1*B*Cl3^i^	0.84(1)	2.50(2)	3.300(3)	161(6)
O1H1*A*O3^ii^	0.84(1)	2.00(2)	2.823(4)	167(5)
O2H2*B*O7^iii^	0.84(1)	2.02(2)	2.853(3)	176(6)
O2H2*A*Cl2^iv^	0.83(1)	2.75(5)	3.347(2)	130(5)
O2H2*A*Cl1	0.83(1)	2.68(4)	3.386(2)	143(6)
O2H2*A*Cl2^iv^	0.83(1)	2.75(5)	3.347(2)	130(5)
O2H2*B*O7^iii^	0.84(1)	2.02(2)	2.853(3)	176(6)
O3H3*A*Cl2	0.84(1)	2.41(2)	3.237(3)	171(5)
O3H3*B*Cl3^v^	0.84(1)	2.71(5)	3.312(3)	130(5)
O3H3*B*Cl4^v^	0.84(1)	2.79(4)	3.512(3)	146(6)
O4H4*B*O1^vi^	0.84(1)	2.01(2)	2.831(4)	167(5)
O4H4*A*O3^vii^	0.84(1)	1.99(2)	2.821(4)	175(6)
O5H5*A*Cl1^viii^	0.84(1)	2.32(1)	3.157(2)	180(6)
O5H5*B*Cl4^vii^	0.84(1)	2.33(2)	3.165(3)	175(5)
O6H6*A*Cl4^viii^	0.84(1)	2.32(1)	3.159(2)	177(4)
O6H6*B*O1^ix^	0.84(1)	1.92(2)	2.754(3)	175(6)
O7H7*A*O2^x^	0.84(1)	1.90(1)	2.739(3)	176(5)
O7H7*B*Cl2^ix^	0.84(1)	2.38(3)	3.181(2)	160(6)
O8H8*A*Cl3^x^	0.84(1)	2.34(2)	3.155(3)	164(5)
O8H8*B*O2^iii^	0.84(1)	1.91(2)	2.738(3)	170(5)
O9H9*A*Cl1^x^	0.84(1)	2.39(1)	3.230(2)	176(4)
O9H9*B*Cl3^xi^	0.84(1)	2.42(2)	3.236(2)	167(5)

Symmetry codes: (i) 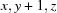; (ii) 
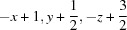
; (iii) 
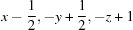
; (iv) 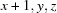; (v) 
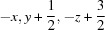
; (vi) 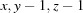; (vii) 
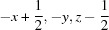
; (viii) 
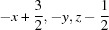
; (ix) 
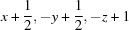
; (x) 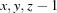; (xi) 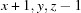.

**Table 4:** Experimental details

	ZnCl_2_2.5H_2_O	ZnCl_2_3H_2_O	ZnCl_2_4.5H_2_O
Crystal data			
*M*_r_	362.66	380.68	434.72
Crystal system, space group	Monoclinic, *P*2_1_/*n*	Triclinic, *P* 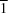	Orthorhombic, *P*2_1_2_1_2_1_
Temperature (K)	150	150	120
*a*, *b*, *c* ()	7.2909(5), 9.7971(5), 15.0912(10)	6.4339(5), 6.5202(5), 14.2769(11)	6.9795(3), 12.5421(6), 18.1849(11)
, , ()	90, 103.375(5), 90	90.910(6), 99.146(6), 95.574(6)	90, 90, 90
*V* (^3^)	1048.72(12)	588.21(8)	1591.86(14)
*Z*	4	2	4
Radiation type	Mo *K*	Mo *K*	Mo *K*
(mm^1^)	5.57	4.98	3.70
Crystal size (mm)	0.27 0.19 0.11	0.60 0.42 0.16	1.00 0.75 0.09

Data collection			
Diffractometer	Stoe IPDS 2	Stoe IPDS 2T	Stoe IPDS 2T
Absorption correction	Integration (Coppens, 1970 ▶)	Integration (Coppens, 1970 ▶)	Integration (Coppens, 1970 ▶)
*T*_min_, *T*_max_	0.287, 0.534	0.093, 0.441	0.050, 0.708
No. of measured, independent and observed [*I* > 2(*I*)] reflections	9997, 2923, 2222	13092, 3239, 3120	40776, 4414, 3955
*R*_int_	0.043	0.091	0.140
(sin /)_max_ (^1^)	0.628	0.693	0.694

Refinement			
*R*[*F* ^2^ > 2(*F* ^2^)], *wR*(*F* ^2^), *S*	0.018, 0.035, 1.01	0.029, 0.089, 1.02	0.021, 0.053, 0.99
No. of reflections	2171	3239	4414
No. of parameters	130	161	208
No. of restraints	15	18	27
H-atom treatment	Only H-atom coordinates refined	All H-atom parameters refined	All H-atom parameters refined
_max_, _min_ (e ^3^)	0.44, 0.36	0.95, 0.95	0.77, 0.64
Absolute structure			Flack *x* determined using 1730 quotients [(*I* ^+^)(*I *)]/[(*I* ^+^)+(*I* )] (Parsons Flack, 2004 ▶)
Absolute structure parameter			0.089(8)

Computer programs: *X-AREA* and *X-RED* (Stoe Cie, 2009 ▶), *SHELXS97* and *SHELXL2012* (Sheldrick, 2008 ▶), *DIAMOND* (Brandenburg, 2006 ▶) and *publCIF* (Westrip, 2010 ▶).

## supplementary crystallographic information

### Crystal data

**Table:**

[Zn(H_2_O)_6_][ZnCl_4_]·3H_2_O	*D*_x_ = 1.814 Mg m^−^^3^
*M**_r_* = 434.72	Mo *K*α radiation, λ = 0.71073 Å
Orthorhombic, *P*2_1_2_1_2_1_	Cell parameters from 33650 reflections
*a* = 6.9795 (3) Å	θ = 1.8–29.6°
*b* = 12.5421 (6) Å	µ = 3.70 mm^−^^1^
*c* = 18.1849 (11) Å	*T* = 120 K
*V* = 1591.86 (14) Å^3^	Prism, colourless
*Z* = 4	1 × 0.75 × 0.09 mm
*F*(000) = 872	

### Data collection

**Table:**

Stoe IPDS 2T diffractometer	4414 independent reflections
Radiation source: fine-focus sealed tube	3955 reflections with *I* > 2σ(*I*)
Detector resolution: 6.67 pixels mm^-1^	*R*_int_ = 0.140
rotation method scans	θ_max_ = 29.6°, θ_min_ = 2.8°
Absorption correction: integration (Coppens, 1970)	*h* = −9→9
*T*_min_ = 0.050, *T*_max_ = 0.708	*k* = −17→17
40776 measured reflections	*l* = −25→25

### Refinement

**Table:**

Refinement on *F*^2^	Hydrogen site location: difference Fourier map
Least-squares matrix: full	All H-atom parameters refined
*R*[*F*^2^ > 2σ(*F*^2^)] = 0.021	*w* = 1/[σ^2^(*F*_o_^2^) + (0.0379*P*)^2^] where *P* = (*F*_o_^2^ + 2*F*_c_^2^)/3
*wR*(*F*^2^) = 0.053	(Δ/σ)_max_ = 0.001
*S* = 0.99	Δρ_max_ = 0.77 e Å^−^^3^
4414 reflections	Δρ_min_ = −0.64 e Å^−^^3^
208 parameters	Absolute structure: Flack *x* determined using 1730 quotients [(*I*^+^)-(*I*^-^)]/[(*I*^+^)+(*I*^-^)] (Parsons & Flack, 2004)
27 restraints	Absolute structure parameter: 0.089 (8)

### Special details

**Table:**

Geometry. All esds (except the esd in the dihedral angle between two l.s. planes) are estimated using the full covariance matrix. The cell esds are taken into account individually in the estimation of esds in distances, angles and torsion angles; correlations between esds in cell parameters are only used when they are defined by crystal symmetry. An approximate (isotropic) treatment of cell esds is used for estimating esds involving l.s. planes.

### Fractional atomic coordinates and isotropic or equivalent isotropic displacement parameters (Å^2^)

**Table:**

	*x*	*y*	*z*	*U*_iso_*/*U*_eq_	
Zn1	0.77065 (5)	0.00714 (2)	0.06201 (2)	0.01250 (7)	
Zn2	0.31706 (5)	0.03464 (3)	0.81771 (2)	0.01309 (7)	
Cl3	0.27768 (11)	−0.08062 (5)	0.91458 (4)	0.01637 (13)	
Cl4	0.22957 (11)	−0.06620 (6)	0.71906 (4)	0.01873 (14)	
Cl1	0.62302 (10)	0.09178 (6)	0.80989 (4)	0.01949 (14)	
Cl2	0.11859 (11)	0.17633 (6)	0.83407 (4)	0.01869 (14)	
O5	0.7005 (4)	0.0248 (2)	0.17173 (12)	0.0221 (4)	
O6	1.0486 (3)	−0.03761 (19)	0.08567 (14)	0.0194 (4)	
O4	0.6863 (4)	−0.15048 (17)	0.05792 (14)	0.0230 (5)	
O7	0.8639 (3)	0.17112 (16)	0.06267 (13)	0.0155 (4)	
O2	0.7609 (3)	0.27476 (16)	0.93613 (13)	0.0180 (4)	
O3	0.0251 (4)	0.25556 (19)	0.66850 (15)	0.0233 (5)	
O1	0.6408 (4)	0.75102 (19)	0.91896 (15)	0.0216 (5)	
O8	0.5001 (3)	0.05893 (19)	0.03098 (15)	0.0216 (5)	
O9	0.8442 (3)	−0.00564 (19)	−0.04927 (12)	0.0193 (4)	
H6A	1.106 (7)	−0.008 (3)	0.1205 (18)	0.027 (12)*	
H1A	0.732 (5)	0.761 (4)	0.890 (2)	0.030 (12)*	
H4B	0.681 (8)	−0.188 (3)	0.0199 (16)	0.031 (12)*	
H7A	0.829 (8)	0.201 (4)	0.0235 (16)	0.033 (13)*	
H9A	0.784 (6)	0.017 (4)	−0.0860 (17)	0.029 (12)*	
H5A	0.747 (7)	−0.006 (4)	0.2084 (19)	0.043 (15)*	
H5B	0.586 (3)	0.032 (4)	0.184 (3)	0.041 (14)*	
H8A	0.433 (6)	0.015 (3)	0.008 (2)	0.031 (13)*	
H7B	0.816 (9)	0.204 (4)	0.099 (2)	0.054 (18)*	
H8B	0.423 (6)	0.106 (3)	0.045 (3)	0.028 (12)*	
H6B	1.069 (8)	−0.1034 (13)	0.084 (3)	0.038 (15)*	
H9B	0.959 (3)	−0.014 (5)	−0.061 (3)	0.050 (16)*	
H4A	0.629 (7)	−0.181 (4)	0.092 (2)	0.035 (14)*	
H2A	0.780 (9)	0.239 (4)	0.8979 (19)	0.049 (17)*	
H3A	0.046 (8)	0.228 (4)	0.7098 (15)	0.040 (15)*	
H1B	0.550 (6)	0.791 (4)	0.906 (3)	0.047 (17)*	
H2B	0.645 (3)	0.292 (4)	0.939 (4)	0.050 (17)*	
H3B	−0.041 (7)	0.310 (3)	0.676 (4)	0.047 (16)*	

### Atomic displacement parameters (Å^2^)

**Table:**

	*U*^11^	*U*^22^	*U*^33^	*U*^12^	*U*^13^	*U*^23^
Zn1	0.01058 (15)	0.01326 (13)	0.01365 (14)	0.00118 (11)	−0.00037 (11)	0.00004 (10)
Zn2	0.00971 (15)	0.01571 (13)	0.01386 (14)	−0.00083 (11)	−0.00053 (11)	−0.00135 (11)
Cl3	0.0161 (3)	0.0176 (3)	0.0154 (3)	−0.0007 (2)	−0.0006 (2)	0.0012 (2)
Cl4	0.0175 (3)	0.0241 (3)	0.0146 (3)	−0.0049 (3)	−0.0008 (3)	−0.0039 (2)
Cl1	0.0108 (3)	0.0288 (3)	0.0189 (3)	−0.0047 (2)	0.0008 (3)	−0.0050 (3)
Cl2	0.0137 (3)	0.0185 (3)	0.0239 (4)	0.0027 (2)	−0.0013 (3)	−0.0032 (2)
O5	0.0198 (11)	0.0340 (11)	0.0126 (9)	0.0063 (10)	0.0007 (8)	0.0015 (8)
O6	0.0161 (10)	0.0193 (10)	0.0227 (11)	0.0046 (9)	−0.0063 (8)	−0.0035 (9)
O4	0.0319 (13)	0.0176 (10)	0.0195 (11)	−0.0053 (9)	0.0026 (11)	−0.0007 (8)
O7	0.0147 (10)	0.0154 (9)	0.0164 (10)	0.0004 (7)	0.0007 (8)	−0.0009 (8)
O2	0.0170 (11)	0.0175 (9)	0.0194 (10)	−0.0004 (8)	0.0005 (9)	−0.0012 (8)
O3	0.0220 (12)	0.0236 (11)	0.0245 (13)	0.0060 (9)	−0.0043 (10)	−0.0005 (9)
O1	0.0189 (12)	0.0199 (10)	0.0260 (12)	−0.0012 (9)	0.0012 (9)	−0.0014 (9)
O8	0.0122 (10)	0.0244 (11)	0.0281 (13)	0.0056 (8)	−0.0062 (9)	−0.0088 (9)
O9	0.0157 (10)	0.0294 (11)	0.0127 (9)	0.0038 (9)	0.0025 (8)	0.0012 (8)

### Geometric parameters (Å, º)

**Table:**

Zn1—O4	2.064 (2)	Zn1—O7	2.157 (2)
Zn1—O6	2.065 (2)	Zn2—Cl1	2.2570 (8)
Zn1—O5	2.066 (2)	Zn2—Cl2	2.2728 (8)
Zn1—O8	2.075 (2)	Zn2—Cl4	2.2783 (8)
Zn1—O9	2.094 (2)	Zn2—Cl3	2.2953 (8)
			
O4—Zn1—O6	90.86 (10)	O6—Zn1—O7	88.54 (9)
O4—Zn1—O5	94.02 (10)	O5—Zn1—O7	87.92 (10)
O6—Zn1—O5	92.91 (10)	O8—Zn1—O7	88.72 (9)
O4—Zn1—O8	91.75 (10)	O9—Zn1—O7	90.25 (9)
O6—Zn1—O8	175.34 (10)	Cl1—Zn2—Cl2	109.67 (3)
O5—Zn1—O8	90.76 (10)	Cl1—Zn2—Cl4	112.35 (3)
O4—Zn1—O9	87.81 (10)	Cl2—Zn2—Cl4	111.95 (3)
O6—Zn1—O9	87.15 (9)	Cl1—Zn2—Cl3	111.20 (3)
O5—Zn1—O9	178.17 (10)	Cl2—Zn2—Cl3	108.60 (3)
O8—Zn1—O9	89.09 (10)	Cl4—Zn2—Cl3	102.86 (3)
O4—Zn1—O7	178.00 (10)		

### Hydrogen-bond geometry (Å, º)

**Table:**

*D*—H···*A*	*D*—H	H···*A*	*D*···*A*	*D*—H···*A*
O1—H1*B*···Cl3^i^	0.84 (1)	2.50 (2)	3.300 (3)	161 (6)
O1—H1*A*···O3^ii^	0.84 (1)	2.00 (2)	2.823 (4)	167 (5)
O2—H2*B*···O7^iii^	0.84 (1)	2.02 (2)	2.853 (3)	176 (6)
O2—H2*A*···Cl2^iv^	0.83 (1)	2.75 (5)	3.347 (2)	130 (5)
O2—H2*A*···Cl1	0.83 (1)	2.68 (4)	3.386 (2)	143 (6)
O2—H2*A*···Cl2^iv^	0.83 (1)	2.75 (5)	3.347 (2)	130 (5)
O2—H2*B*···O7^iii^	0.84 (1)	2.02 (2)	2.853 (3)	176 (6)
O3—H3*A*···Cl2	0.84 (1)	2.41 (2)	3.237 (3)	171 (5)
O3—H3*B*···Cl3^v^	0.84 (1)	2.71 (5)	3.312 (3)	130 (5)
O3—H3*B*···Cl4^v^	0.84 (1)	2.79 (4)	3.512 (3)	146 (6)
O4—H4*B*···O1^vi^	0.84 (1)	2.01 (2)	2.831 (4)	167 (5)
O4—H4*A*···O3^vii^	0.84 (1)	1.99 (2)	2.821 (4)	175 (6)
O5—H5*A*···Cl1^viii^	0.84 (1)	2.32 (1)	3.157 (2)	180 (6)
O5—H5*B*···Cl4^vii^	0.84 (1)	2.33 (2)	3.165 (3)	175 (5)
O6—H6*A*···Cl4^viii^	0.84 (1)	2.32 (1)	3.159 (2)	177 (4)
O6—H6*B*···O1^ix^	0.84 (1)	1.92 (2)	2.754 (3)	175 (6)
O7—H7*A*···O2^x^	0.84 (1)	1.90 (1)	2.739 (3)	176 (5)
O7—H7*B*···Cl2^ix^	0.84 (1)	2.38 (3)	3.181 (2)	160 (6)
O8—H8*A*···Cl3^x^	0.84 (1)	2.34 (2)	3.155 (3)	164 (5)
O8—H8*B*···O2^iii^	0.84 (1)	1.91 (2)	2.738 (3)	170 (5)
O9—H9*A*···Cl1^x^	0.84 (1)	2.39 (1)	3.230 (2)	176 (4)
O9—H9*B*···Cl3^xi^	0.84 (1)	2.42 (2)	3.236 (2)	167 (5)

Symmetry codes: (i) *x*, *y*+1, *z*; (ii) −*x*+1, *y*+1/2, −*z*+3/2; (iii) *x*−1/2, −*y*+1/2, −*z*+1; (iv) *x*+1, *y*, *z*; (v) −*x*, *y*+1/2, −*z*+3/2; (vi) *x*, *y*−1, *z*−1; (vii) −*x*+1/2, −*y*, *z*−1/2; (viii) −*x*+3/2, −*y*, *z*−1/2; (ix) *x*+1/2, −*y*+1/2, −*z*+1; (x) *x*, *y*, *z*−1; (xi) *x*+1, *y*, *z*−1.
